# Engaging the agricultural community in the development of mental health interventions: a qualitative research study

**DOI:** 10.1186/s12888-023-04806-9

**Published:** 2023-06-05

**Authors:** Emma King, Kate Lamont, Charlotte Wendelboe-Nelson, Chris Williams, Cameron Stark, Hugo C van Woerden, Margaret Maxwell

**Affiliations:** 1 University of Stirling, Scotland, United Kingdom; 2 Scotland’s Rural College (SRUC), Scotland, United Kingdom; 3 University of Edinburgh, Scotland, United Kingdom; 4 Glasgow University, Scotland, United Kingdom; 5 University of the Highlands and Islands, Scotland, United Kingdom

**Keywords:** Mental health, Suicide prevention, Farming, Qualitative research

## Abstract

**Background:**

Farmers and those involved in the wider agricultural industry have a high suicide rate. They are also a ‘hard to reach’ group who make less than average use of mental health services. There is therefore a need to understand how best to develop interventions that meet their needs. The aims of this study were to develop a deeper understanding of the farming context and target population and to engage farmers in the shaping of two potential mental health interventions that could be incorporated in a pilot RCT.

**Methods:**

The study was informed throughout by a reference group, who assisted in co-production of the research materials. A snowball approach was used to recruit interested individuals who had an association with farming. Twenty one telephone interviews were undertaken and analysed using the six phases of thematic analysis proposed by Braun and Clarke.

**Results:**

Key themes (and sub-themes shown in brackets) related to the study aims were: everyday life (work-life balance; isolation and loneliness); farm management (technology and social media; production, people management, learning and teaching; external pressures; livestock and farm production; financial aspects); demographics (effects of aging); engagement (appropriate wording when talking about mental health; recognising need for help; religion; normalising mental health issues; approaching the conversation); training (mental health training for supporters of the farming community; health & safety and the inclusion of mental health training); and personal stories and experiences, which was an emerging theme.

**Conclusions:**

Recruiting farmers into research studies is best done by meeting farmers where they are found, for example, farmers marts. Accessibility of content, tailoring to the farming community, and guided support are key to effective recruitment and retention.

**Supplementary Information:**

The online version contains supplementary material available at 10.1186/s12888-023-04806-9.

## Background

Farming, including the wider agricultural industry, has one of the highest rates of mortality of any industry [1], accounting for 1.5% of the British economy but 24% of all workplace deaths [2]. As well as work-related accidents, depression in farmers is increasing and suicide rates are the highest in any occupational group [3, 4]. In 2019-20 133 people involved in UK agriculture took their own lives (ONS). Farming has higher rates of suicide than the national average and other rural males [1], with the risk of suicide in specific agricultural roles such as crop harvesting and rearing animals almost twice the national average. Farmers are also more likely to report thinking that life is not worth living [3, 4]. The problem of poor mental health in farming is not unique to UK, with reports of health issues, burnout, anxiety and depression etc. in Nigeria, Finland, Norway, Canada, and Australia [5]. Two useful reviews have also been undertaken, focusing on north America [6, 7]. Four out of five farmers in Scotland under the age of 40 consider mental health to be the biggest problem facing the agricultural community [8].

Farmers have been found to have lower rates of health seeking behaviour for mental illness than the general population, and suicide in farmers may be more likely to be impulsive [3]. Although, despite the conclusion that nearly all farmers who took their own life showed clear intent, suicide in male farmers has also been found to be an end point to a series of difficulties that have built up over time rather than a reaction to an immediate crisis [1].

Higher rates of suicide may therefore be due to easier access to lethal means and a more pragmatic view of death [1]. Farmers are more likely to have access to firearms, although changes to the law for gun ownership in England and Wales in 1989 means hanging has now taken over as the principle method of suicide [1].

### Mental health pressures on the agricultural community

Farming has a unique mix of intertwined circumstances that are potentially hazardous to mental health, such as social isolation, long hours, variable income due to circumstances out with one’s control such as the weather, [1, 9]. Stress is linked to depression and high level of stress have been observed in farmers [10]. Compared to the general population, Norwegian farmers were more likely to report that life was not worth living [9].

### Isolation and loneliness and rurality

Farmers and agricultural workers both live and work almost exclusively in rural areas, which is almost unique amongst occupational groups [1, 3]. Social and geographical isolation has been found to impact on mental wellbeing in farming communities, with many farmers experiencing isolation and loneliness [3, 11]. Rural areas in general suffer from geographical isolation, lower levels of educational attainment, worker shortages, young people moving away, and difficulty accessing appropriate healthcare [5, 12]. Social support, including emotional and practical support, is a protective factor in countering stress [9]. Not having social support or anyone to confide in was a large factor in farmer suicide [3, 13].

### Agricultural incomes and housing

Farm income in several countries has been dropping since 2013/14 with many farmers experiencing high stress levels due to financial challenges [3, 5, 10]. The average income from a 500 hectare farm in the UK fell from £80 000 in 1995/1996 to £2500 in 2000/2001 and less than 50% of farmers currently make a living from farming [1, 11]. Financial stress is exacerbated by the reliance on unpredictable factors such as the weather and crop/animal disease [3]. Housing is a big concern to agricultural workers and tenant farmers as housing is often tied to insecure jobs and social housing is more scarce in rural areas [3].

Farmers in the UK with farms of < 300 acres are more likely to take their own lives, suggesting that those with smaller farms suffer more stress and have less support [3]. The biggest cause of distress was unemployment, independent of financial status [13]. A discrepancy between actual income and financial aspirations was strongly related to wellbeing [9].

### Family, community and social cohesion

Most farms continue to be business owned and operated within one family, meaning work, home, and family roles are often blurred [1]. Tensions can exist between different family members working on the farm, which can be exacerbated by a lack of clear succession planning [1, 11]. The farm is often also the family home and may be shared between different members of the family, making it difficult for people to extract themselves from the business. Farmers also face societal pressure to maintain a farm that has been handed down for many generations and that they in turn hope to hand to their children [9]. Farmers cite passing the farm onto their children as a reason for investing in improvements or expensive automation [9]. In summary, although the farming community can be tight-knit there is also ongoing competition from neighbouring farmers [5].

### Physical demands and working hours

Farming is physically demanding work and farmers work long hours in a variety of weather conditions [1, 3, 5]. The drop in farm income is making it more difficult for farmers to employ additional labour, adding both to isolation and farmers having to manage physical work alone [9]. The larger the herd the lower the job satisfaction [9]. Farmers often find it difficult to take holidays [3]. Farmers also suffer long-term exposure to pesticides and other chemicals, which may be linked to health conditions including problems with the central nervous system [1, 3]. One third of farmers in the UK report physical health problems that are serious enough to interfere with work and experiencing long-term back pain was a predictor of suicide [3]. Increased alcohol use also increases the likelihood of mental distress [3, 13].

### Unpredictable environmental factors

Whilst farmers experience a complex relationship with the uncontrollable nature of the weather and working outdoors there are also benefits from the resilience that farming brings and the benefits of farm work keeping them active [14]. Exposure to natural landscapes can be seen as a source of relaxation and prevention of mental health issues but this also brings with it the unpredictability of weather and distance from services [5, 12], and may be less preventative in an occupational setting [15].

### Regulation and bureaucracy

New technology, regulation, administration, and digitalisation bring challenges for mental health [5, 11, 14]. Coping with paperwork was ranked as one of the highest stressors for farmers, who perceive a lack of support dealing with bureaucracy [3, 14]. ’Technostress’ has been used to describe the problem of having to adapt to new information and communication technology (ICT) [9]. Whilst older farmers may experience more stress of new ICT and automation, they are also more likely to have more expertise in traditional methods [9].

### Gender and age

Depression in farmers increases with age and is more associated with males [16]. Males in farming could be influenced by traditional expectations of male roles within the family and wider community, as well as men being less likely to socialise with close friends outside their family [1]. Men are more likely to link their wellbeing to financial success, whereas women focus more on relationships and work-life balance [9].

Older male farmers are much less likely to access help for their mental health than women or younger farmers, and rural clinicians found wives and children were more likely to encourage older male farmers to access help [5]. As with older farmers, in a wide ranging review, mental health consultation rates for rural young women have been found to be double those for rural young men, and rural young men are less likely to seek mental health help from their GP compared to urban young men [16].

Despite reporting lower levels of depression, rural men are much more likely to take their own lives than women, which has been described as a ‘silent crisis’ [4]. Men are more likely to be concerned about stigma around mental health and farmers are more likely to enact more traditional views of masculinity such as stoicism and self-efficacy.

Women in farming have different stressors. They have reported that they sometimes have to take on more work outside the farm to bring in additional income as farming incomes drop, and take on both the stress of the farm and additional stress of managing the wellbeing of family members [1]. Women are also likely to take on the bulk of the domestic work, despite also working outside the home and on the farm [1, 9].

Mental health disorders are more prevalent in youth than at any other time in life [13]. Children and young people living in farming communities face similar pressures to other rural young people, with additional stress that is unique to farming [1, 9]. Rural young people face greater challenges than those in urban areas in accessing appropriate services and doing so confidentially and without stigma [13].

#### The gap in current mental health provision

Living in rural areas may make it difficult to access help for mental health needs due to a variety of factors, including lack of awareness of services available, cost of travel, lack of privacy, no local mental health services, long travel times, lack of choice of health providers, increase reliance on local GPs [1, 3, 5, 16]. Clinicians often lack training around farming as a sub-culture, for example that farmers cannot take extended periods of time off work or the difficulty of separating work and home life [3]. Farmers are often reluctant to seek help so clinicians must be aware that conversations might occur indirectly whilst they are consulting a doctor for another reason [9]. Seasonal farm workers often lead a transient lifestyle that makes it difficult to access continuity of care [1, 5].

Whilst the independence exhibited by farmers can be a positive factor in mental wellbeing, it can also be a barrier to seeking help [1, 5]. Stigma can also play a negative role in farmers accessing mental health help, with depression being seen as a ‘weakness of character’ [1, 16]. As farming communities are small, this increases the visibility and makes it more difficult for people to access help without the fear of stigma, compared to those in more urban areas [1, 3, 10].

Farmers reported believing that professional mental health help would be inadequate with long waiting times [16]. Instead, social and community connections are particularly important in rural communities and farmers are much more likely to turn to their own community for help [5, 12, 13, 17]. Farmers are more likely to talk to others in the farming community and respected individuals such as vets [18]. This could be a useful tool in educating farmers to recognise, and provide support for, mental health problems within their own community [5].

Although farming is an occupation that may contribute to high stress and poor mental health there are also positives to be taken from farming communities, such as high resilience, time spent outside, close relations, shared identities, and community cohesion [5, 9].

The UK Department for Environment, Food, and Rural Affairs (Defra) define wellbeing as ‘a positive physical, social and mental state; it is not just the absence of pain, discomfort and incapacity’, but wellbeing can be different for different individuals [9]. A recent report on farming and mental health in Wales identified a three-pronged approach of preventing uncertainty, protecting farmer’s mental health against the impact of ongoing challenges, and promoting mental health and well-being amongst farming communities [11]. The Welsh report also recognized four areas for targeting mental health: *(i) raising awareness about mental well-being and support targeted to the farming sector, (ii) increasing mental health literacy amongst support agencies, (iii) partnership working in order to integrate mental health and well-being across farm facing services, and (iv) outreach programmes*. However, none have been identified that evaluated to understand the acceptability and efficacy [11].

As farmers are more likely to turn to each other for support, using the existing farming network could be a key target for mental health support [13]. Farmers report the most benefit from chatting to others who understand the farming life [9, 10]. Other farming specific approaches identified have included promotion in farming media, educating younger people in the farming community, and targeting mental health advice, support, and counselling to rural areas, along with other existing networks such as sports clubs, training providers, religious groups [3, 13]. Farmers are more likely to seek help from their own communities or from members within their communities (such as vets) or to use anonymous support such as the internet or self-help booklets [18].

Cognitive behavioural therapy (CBT) is a psychological therapy with the best established evidence for the management of common mental health problems [19]. CBT has advantages in that it can be completed in a relatively short period of time compared with other talking therapies; its structured nature means it can be provided in different formats (including remote and self-directed formats); and it teaches practical strategies that can be used in everyday life. Interactive computerised CBT has been found to be acceptable in U.S. rural communities in relation to privacy, accessibility, user-friendliness and cultural appropriateness [20].

There is also growing evidence from other areas that self-help interventions can be a low-cost and wide-reaching method of delivering behaviour change information, whilst reducing travel time and stigma (Emma paper). Interventions delivered by SMS text messages have been used to target hard to reach groups, such as those from deprived areas, heavy drinkers, or illicit drug users. Behavioural targets have included smoking cessation, binge drinking, weight loss, physical activity, and medication adherence [21–23]. However, there is no current knowledge concerning preferences and acceptability or up-take of remote interventions by farmers in the UK.

This study was undertaken in Scotland. The south of the country generally contains more fertile, larger farms than in the north of the country. Many farmers and agricultural workers live on small holdings, and have diversified to obtain more than one source of income, for example, by having a part time job, which supplements their farming income.

### Aim and research questions

The focus of this qualitative study was to inform a pilot RCT of two mental health interventions: a Cognitive Behavioral Therapy package “Living Life to the Full for Farmers” (LLTTFF) and secondly a social and emotional support service that includes a telephone helpline, which is staffed by mental health first aid trained staff and volunteers from the Royal Scottish Agricultural Benevolent Institution, delivered separately or in combination as a psycho-social intervention. Living Life to the Full is based on a long standing program of research, which has subsequently been tailored for different audiences [24]. The helpline grew out of the farming community and was experientially developed over time, based on the experience of staff.

The two study aims were to:

- Understanding the farming context and target population.
- How best to engage farmers in a mental health intervention.

The aims linked to two broad sets of research questions:

1. What are the preferences of farmers regarding remote/anonymous support for their mental health? What do interventions designed for farmers look like and how should they be delivered?
2. What are the barriers and facilitators for reaching people from the farming community to engage them in an intervention to improve mental health?

## Methods

The study was informed throughout by a reference group, who assisted in co-production of the research materials. The group included farmers, an agricultural consultant, academics, veterinary and human health clinicians, along with representatives for both the interventions and from the Scottish Rural Mental Health Forum. The process of co-production involved, enabled us to balance the different perspectives and priorities of different group members, to achieve an approach which was simultaneously academically robust and ‘farmer friendly’. As an example, the reference in any recruitment. The group advised on how best to reach farmers with fliers / leaflets etc. and they also advised about images used in written materials.

### Recruitment and sample selection

We used a ‘snowball approach’, inviting individuals from the farming community, and individuals who come into contact with people from the farming community in Scotland, to recruit participants who would share their views and suggestions about the language and methods that should be use to attract and engage with individuals in the agricultural community, who might be experiencing common mental health problems, such as anxiety or depression.

One focus group was held, which was conducted online due to COVID-19 restrictions. Telephone interviews were undertaken as alternative to face-to-face interviews for COVID-19 related reasons, providing a final sample of 21 individuals who were interviewed over the phone.

#### Ethical approval

**Fig. 1:**
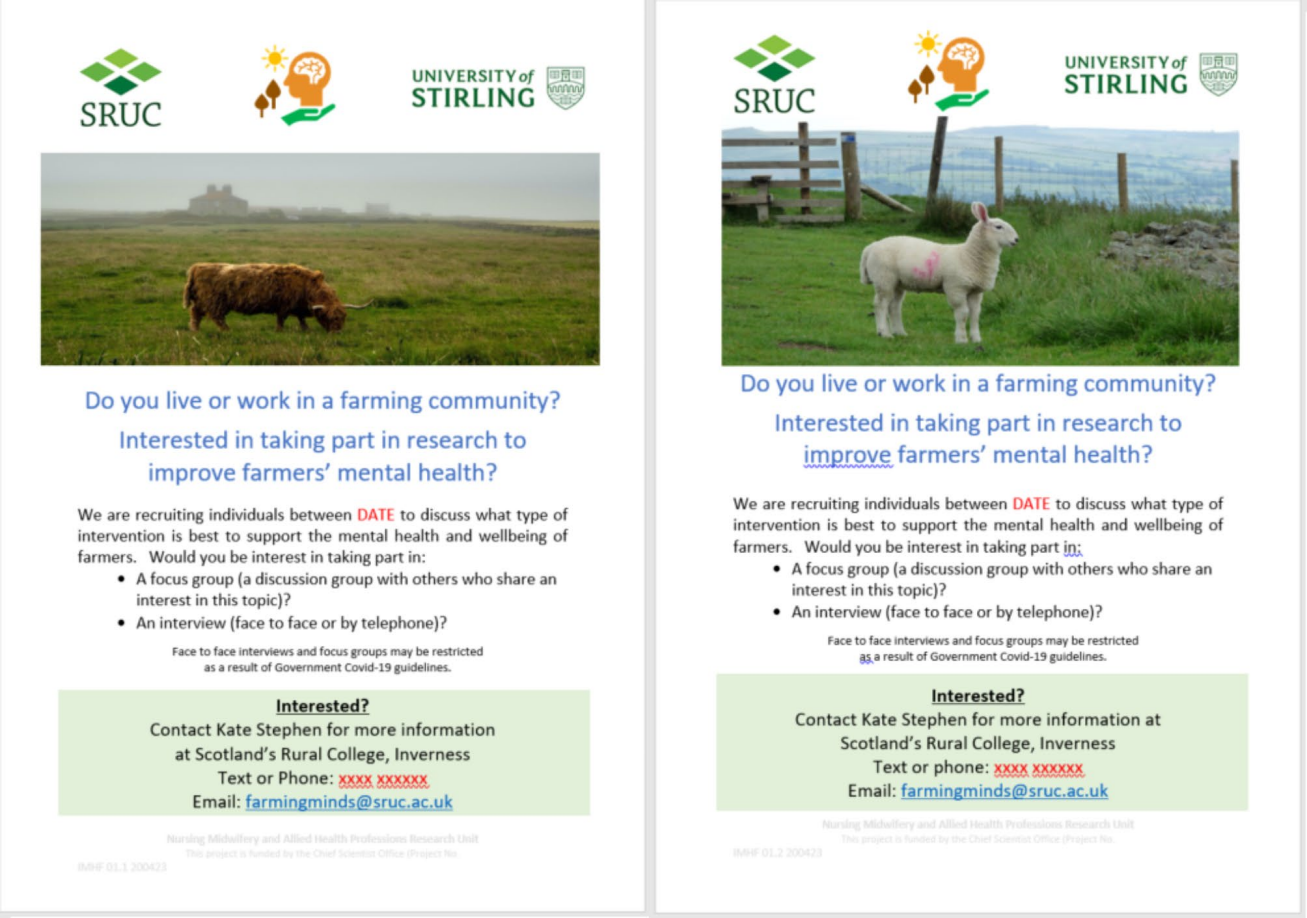
Examples of first cycle recruitment materials

was obtained from the University of Stirling General University Ethics Panel (GUEP) and approval received in May 2020. Recruitment materials were produced (Fig. 1) along with ‘Participant Information Sheets’ and ‘Consent Forms’, all of which were available in electronic and hard copy.

Information sheets and consent forms were emailed or posted ahead of the interview. The phone interviews followed a semi structured format, aiming to broach a range of topics and questions based on a topic guide (see Supplementary Materials), while allowing the interviewee to talk freely and in an unstructured manner if they so wished. The topic guide was developed in consultation with the reference group and in line with the wider academic literature. Before proceeding, the principles of confidentiality and the process of audio-recording was explained to participants, and informed consent obtained. Interviews lasted between 20 and 60 min and were audio-recorded. All personal identifiers were removed after transcription. Details which could be used to identify individuals from the community in which the individual lived were not utilised.

One on-line focus group was held with the UK National Farmers Union Regional Managers. This focus group was not audio recorded but notes were taken for analysis. Focus group participants were shown a range of sample leaflets and asked for feedback about the content and for their views about where these should be distributed.

The transcribed interviews were analysed using Thematic Analysis (TA), as developed for use in qualitative psychology [25]. Our aim was to identify views and opinions of people within the farming community, in relation to acceptable ways of reaching individuals with support options for their mental health. For that reason, we chose to analyse the collected interview data using an inductive TA approach, identifying themes at an interpretive level from a constructionist perspective. With minor adaptations, our analysis follows the six phases proposed by Braun and Clarke : familiarisation with data, generation of initial codes, searching for themes, reviewing themes, defining and naming themes, and reporting of the results.

#### Familiarisation with the data

The data was transcribed and entered into NVivo 12. Initial familiarisation was led by CWN. The initial codebooks were discussed and compared, to generate an initial set of questions guiding the coding.

#### Generating initial codes

The final coding of all 21 interview transcripts was conducted by CWN, EK, and KL, following the generated set of guiding questions. Interesting features were identified across all transcripts and data relevant to each code was collated.

#### Searching, reviewing, defining and naming themes

Individual approaches to coding were reviewed at a project group meeting, and collated into initial themes. During this process, six potential themes were chosen: everyday life and its impact on MH; MH training; the effects of age on MH; farm management and MH; and personal experience and real-life stories. During the ongoing analysis the specifics of each theme were refined and the codes were further collated into subthemes within each main theme. Clear definitions for each theme and subthemes were chosen.

#### Reporting of the results

A final analysis was conducted, involving the selection of extracts and relating them back to the research question based on:

- The overall research questions driving the project.
- The questions the participants responded to most during the interviews.
- The set of questions that had been developed to inform the coding process.

## Results

### Recruitment and participant characteristics

A total of 21 interviews took place during 2020-21. Demographics of interviewees are shown in Table 1. ‘Farmer’ refers to farmers as well as crofters, part-time and fulltime. ‘Other’ refers to any other profession that interacts with the farming community on a regular basis (e.g. veterinary, consultant, salesperson, lawyer, banker etc.)

**Table 1:** List of participants

Interviewee	Gender	Profession
1FM01	Female	Farmer
1FM02	Male	Other
1FM03	Male	Farmer
1FM04	Male	Other
1FM05	Male	Farmer
1FM06	Male	Other
1FM07	Male	Other
1FM08	Male	Farmer
1FM09	Male	Other
1FM10	Male	Other
1FM11	Male	Other
1FM12	Male	Other
1FM13	Female	Farmer’s wife
1FM14	Female	Other
1FM15	Female	Farmer’s wife
1FM16	Female	Farmer’s wife
1FM17	Male	Farmer/Other
1FM18	Male	Farmer/Other
1FM19	Female	Other
1FM20	Male	Other
1FM21	Male	Other

### Outcomes of thematic analysis

The main themes and subthemes generated from the thematic analysis are described in Table 2 and detailed below. In line with the GUIDED checklist for developing interventions our results are structured into two main themes:

- Understanding the farming context and target population (GUIDED items 1 & 3).
- How best to engage farmers in a mental health intervention (GUIDED item 8).

An emergent theme of personal stories and experiences was also included, which overlapped with both of the above main themes.

**Table 2:** Main themes and sub-themes

Study aim	Initial questions guiding the coding	Themes	Subthemes
Understanding the farming context and target population	How can everyday life in a farming community affect mental health?	Everyday life	Work-life balance
			Isolation and loneliness
	How does the daily management of the farm affect mental health?	Farm Management	Technology and social media
			Production, people management, learning and teaching
			External pressures
			Livestock and farm production
			Financial aspects
	How does age affect the way individuals in the farming community look at mental health?	Age and gender	Effects of aging
How best to engage farmers in a mental health intervention	What are the best ways of reaching/engaging people in the farming community with support options for their mental health?	Engagement	Appropriate wording when talking about mental health
			Recognising need for help
			Religion
			Normalising mental health issues
			Approaching the conversation
	What types of mental health training is appropriate to the farming community?	Training	Mental health training for supporters of the farming community
			Health & safety and the inclusion of mental health training
		Personal stories and experiences of what can help (emergent theme)	Who: Individuals, organisations, & companies
			What: Examples
			How: Case studies
			Anecdotes

The findings are expanded on below.

#### Theme 1: everyday life

##### *Theme 1.1: work-life balance*

The main difficulty highlighted by respondents was the lack of work-life balance for farmers. This often led to difficulties being able to ‘switch off’ or get away from the farm, because it was often their home as well as their job.

“*You can relate their poor mental health to their business and actually - it’s very often the case that a farmer’s farming life is not a business, it’s their life. They’re not doing it, they’re living it. Farming is a lifestyle.*” (1FM02. Male. Other).

We found, in many cases, that farmers are likely to focus primarily on looking after the livestock and upkeep of the business side of the farm. Looking after themselves and their own health comes second. When it comes to neglecting the livestock, things in their private life had been neglected for a long time and help should have come a lot sooner:

*“And then if you do it in another way where there’s quite a few bachelor farmers, are they eating properly, you know, that’s an observation, and tidiness when you do go into a house, and I am old school, it’s how tidy is the farm? That’s another observation, and then the livestock. So there’s a culmination of quite a few things that you can try and do and help that takes the pressure of them.” (1FM12)*.

When giving examples of different issues and challenges, it was important to some interviewees to make it clear they were not talking about their own farm or their own situation:

*“Whereas a traditional male thinks they’ve got their job and that’s it, now I’m painting a pretty bleak picture, I’m not just saying that’s not exactly what happens in my experience but there is a tendency for that to happen.” (1FM15)*.

##### *Theme 1.2: isolation and loneliness*

A key issue identified in the transcripts was ‘loneliness’. Loneliness in relation to work but also in relation to life in general; with the two (work and life in general) being very difficult to distinguish between:

*“Personally I think you’re missing the point. And the point is that farmers are lonely. Very, very lonely just now. I mean, particularly with COVID just happening, I find that in my line of work I’m having less contact with my farmers and they’re wanting to have more contact with me just simply because there’s nobody coming up the farm drive anymore.” (1FM06)*.

Respondents commented that an app-based or online mental health intervention was not going to address the underlying problem, which was that many famers needed more social interaction and somebody to talk to.

#### Theme 2: farm management

##### *Theme 2.1: technology and social media*

Whilst the younger generation of farmers used technology more often there was a recognition that many older farmers were not computer literate, and that rural areas often had poor broadband connections. Farmers might have no access to email or video calls, and this was becoming more of a struggle as much of the paperwork now needs to be completed online.

*“And then just IT literacy, so many of the folk that I came into contact with in the office was when staff mainly went online and they [the farmers] weren’t online at all, they didn’t have a computer at home so they just wanted to come in and get help with the paper form, or that was the only time they used the computer was when you sat with them and done their SAF online with the computer in the office.”* (1FM14).

##### *Theme 2.2: production, people management, learning and teaching*

One interviewee in particular highlighted the lack of training and knowledge of farmers to effectively manage staff, which in turn could lead to lack of wellbeing for farm workers.

*“Farming people aren’t people persons […] most people or most farmers are farmers because they have a passion for growing crops or they have a passion for livestock. Really it should be turned around the other way because […] the best way to get results is first by making the most of the people that work for you because they are your biggest asset that you’ve got.”* (1FM16).

Managing staff could also be stressful for farmers, and respondents reported that having good staff and being able to manage them well made a huge difference to people’s wellbeing.

*“So if the staff are ticking, if they’re good and they’re producing the goods at the end of the day his job is easier to run, it’s when things go horribly wrong and you’ve got to juggle it and you’re not in your comfort zone, that’s when perhaps you do need a different set of mental health skills, but if you can keep your staff right it makes all the difference.”* (1FM16).

Training for new farmers also didn’t include much training on people management.

*“I was talking to my niece who did a degree at [University] three years ago now and she said, you know, ‘we did very little on marketing, we did very little on wellbeing of people and how to manage people, it’s still basic agriculture’.”* (1FM16).

##### *Theme 2.3: external pressures*

Interviewees saw it as problematic that the public didn’t recognise the difficult working situations that farmers endure. Respondents also expressed the ongoing concerns of external pressures such as Brexit, the importing of food making local prices collapse, and media coverage about climate change and veganism seen to be giving farmers a bad reputation.

*“So to me that’s the, you know, yeah the long hours and the bad weather and the isolation is tricky but that on top of the poor public perception or poor public understanding, to me I think that’s the key, so it’s educating everybody else.”* (1FM15).

##### *Theme 2.4: livestock and farm production*

Livestock were seen as one of the first signs that a farmer may be struggling to cope, and fellow farmers are likely to be the first to notice this.

*“Perhaps the cattle weren’t bedded up as well as they normally would, they weren’t looking as good nick, you know, things weren’t as tidy as they were and he was picking up on people which he thought were struggling mentally.”* (1FM17)

By the time organisations are aware of the situation the farmer has often been struggling for some time.

*“But the stock thing, that takes a wee bit of time for that to happen and that’s the real difficulty, that’s the one that, like, once we see it obviously it’s straightforward to try and do something about that, but the bother is you’re too far down the line then.”* (1FM09)

The uncertainty of stock and of tending crops makes it difficult for farmers and farm workers to attend meetings or be involved in hobbies outside the farm.

*“If you’re a livestock farmer with one employee and that employee has to go off because he’s got a meeting, ’well can’t you do it in the evening or d’you have to go because I really can’t do this job on my own and it’s going to rain tomorrow’ or ‘the cows need milking’ you know, oh dear, there’s no room and it won’t take much for them to tweak. I don’t know but I don’t hear many farming employees with hobbies?”* (1FM16)

##### *Theme 2.5: financial aspects*

Interviewees reported a variety of financial concerns felt by farmers, including small profit margins, reliance of families on farms to provide income and family homes, large debts, and dependency on unstable factors such as weather or livestock health.

*“This farmer doesn’t like it when an agent is required because that costs money. This farmer works a colossal amount of hours and the margins are small. If farming was more profitable, it would be easier to afford an agent when required and it would be possible to afford to pay for someone to cover for a couple of days to get some time off. Currently this farmer doesn’t get time off.”* (1FM03)

A vicious cycle around mental health and finance was reported, with farmers finding it difficult to face financial issues, which in turn led to more stress and anxiety, and small profit margins making it difficult to employ help or take time off. Farmers were also reported as unlikely to discuss financial issues with other farmers.

*“Looking at and getting their paperwork up to date cause that sometimes can be… mail unopened just thrown in a corner and things just getting on top of them, so one of the things we’ve done here is send my secretary and get all the mail, sorted everything all out, VAT is something that they get behind with; it’s little things like that and that helps break the ice.”* (1FM12).

Practical help was highlighted as a key intervention to improve wellbeing, particularly around the paperwork farmers are faced with.

#### Theme 3: age and gender

Farming was acknowledged to be a profession with many older males, who are not used to discussing mental health as freely as the younger generations were seen to be.

*“I mean, farming is an older person’s profession/occupation, whether farmers like to think that or not, you look at the statistics and the average age of a farmer in the UK is… I think it might even be up over 60 now, and people of that generation are of a certain mindset when it comes to talking about feelings and particularly with a certain stigma that’s attached to mental illness.”* (1FM04).

The Young Farmers association was seen to do a lot to promote discussion of mental health, and it was felt that younger generations were used to discussing mental health more freely. Young generations were also seen to be more concerned about their work-life balance and not spending all their time on the farm.

*“A lot of the younger generation going into to farming say, ‘I want to take on the farm but I see my parents life and how stressed they are and their life is 24/7 farming and I’m worried about that happening to me. The concept of work / life balance, of family is more important to them than it has been in previous generations.” (1FM10)*.

Gender was also mentioned, talking about how difficult men often find it to discuss mental health and often clam up. Women felt that they had to think about things beyond the farm and they didn’t get fixated on the job, which was seen to be unhealthy.

“*It’s a traditional male thing as well, you know, as working women we have had to do our work to the best of our ability but we’ve also, […] you’ve got the laundry, you’ve got the food, you’ve got the dog to walk, you’ve got the family to keep up with, you’ve got birthday presents to buy, you’ve got all this, so you learn to prioritise, as a female in a traditional environment you learnt to prioritise a little bit better so you’re not so likely to get bogged down in any one particular area because you’ve got other things to think about.” (1FM15)*.

#### Theme 4: engagement

A common feature for all interviews was the tendency to revert to one topic in particular: engagement; how do we reach people in the farming community with support options for their mental health. ‘Engagement’ was therefore the theme most often mentioned during the interviews.

A key issue in terms of support for the farming community is how to reach and engage the individuals who would benefit from MH support. When analysing the collated data extracts within the ‘engagement’ theme, it was decided to divide them into four subthemes: approaching the conversation, normalising MH issues, recognising need for help, appropriate wording when talking about MH. The coded extracts within each of the subthemes were viewed as elements of engagement; a means to facilitate engagement and further conversation surrounding MH issues in the farming community, underpinning the decision to create subthemes, supporting different aspects of engagement:

“*Yeah, I think my view is there’s two distinct elements to that and the first is reaching them and the second is encouraging them to open up, and I think they’re very different things.*” (1FM04).

##### *Theme 4.1: appropriate wording when talking about mental health*

Interviewees were asked how they felt it would be most appropriate to approach farmers regarding their mental health. The consensus was that farmers would be reluctant to discuss mental health directly, so taking an indirect approach was more favoured.

*“Yeah, ‘how are you getting on with things?’ you know, or I would say ‘is life getting on top of you?’ or ‘is there anything I can do to help with a situation?’”* (1FM06).

*“I think the best phrase is ‘are you coping?’”* (1FM09).

*“For some, it might be a turn off but for others not. For me it depends on my audience I don’t always talk in terms of mental health, I talk in terms of- struggling, struggling with wellbeing, not being in a good place, feeling low, feeling distress – or I talk about stress – because if you are under prolonged stress…….I talk about stress being an normal and natural part of life but too much stress and prolonged exposure to high levels that we can’t cope with is ……….so sometimes you come at it from that angle rather than talk about mental health.”* (1FM10).

Words such as ‘struggling’, ‘anxiety’, ‘coping’, ‘resilience’ were deemed more acceptable than ‘depression’ or ‘mental health’. Farming related terms like ‘bogged down’ or ‘looking after the top paddock’ were also suggested.

However, one respondent did feel that a direct approach could also work well.

*“I think the benefit, if you can call it that, of mentioning suicide is that it puts the focus on farmers to realise the very real and very serious consequences of not having early intervention, and I suppose the risk of focusing purely on positive elements and not mentioning any of the serious consequences is that some people might under-estimate the importance of it. […] What it will also probably do is it will probably resonate with a lot of farmers who know somebody who has committed suicide and if they can make that link between early intervention in mental health and suicide then that might encourage them to get involved, you know, sort of thinking ‘oh my good friend Jim committed suicide therefore it can be no bad thing to try and stop that happening’, whereas if you just focus on the early intervention and the positive that can bring they might not quite make the bridge between the friends that they know who have committed suicide and the benefits of getting involved in this scheme.”* (1FM04).

##### *Theme 4.2: recognising need for help*

During the analysis process a difference was identified between the responses from some farmers and Individuals Related to the Farming Community. In some cases, there was an element of such individuals believing they know what the farmers want and need, and would be able to elicit the information:

“….*it’s a bit of experience I suppose. I just know if somebody’s really down and then what I do is I make sure that all his neighbours go and visit him, you know, I phone up all his neighbours or folk that I know know him and just say ‘keep your eye on so and so, I don’t think he’s in the best of places’ and between us we get there. Between us we get there.*” (1FM06).

“…*that’s what I mean where you go off at tangents and talk about things that are completely alien to what you’re trying to find out and then you just quietly work, it’s a bit like fishing isn’t it, you just quietly bring the net in and then you can summarise the true situation within the business.*” (1FM12).

“*In a situation like that, I’ll quite often revert to a third party, even if it’s slightly false – “I was speaking to a man in Inverness, life’s getting him down – I said I’d get a phone number for him that he can phone to help him feel a bit better”*” (1FM02).

This sentiment of ‘knowing what is right for the farmer’ as a means of approaching the conversation and facilitating engagement is not necessarily reflected as acceptable or desirable in the farmers responses:

“….*possibility of a local farmer as a ‘go to’ person for help, I do not like the idea of an interfering person. A ‘go to’ farmer would be perceived as someone who likes to interfere and know someone else’s business.*” (1FM03).

On occasions it was clear that the interviewed farmer felt uncomfortable when asked about MH and would retract and end the interview in a (more or less) subtle way:

“*I’ve been speaking to you and the boy’s just let out the sheep I was wanting to take a photograph of so I’m afraid I’m going to have to go at the moment, sorry about that.*” (1FM08).

The notion of supporting your neighbours and local community by being the one initiating the conversation surrounding MH seemed difficult for some farmers, and reaching out was also brought up as troublesome:

“*….I would not feel comfortable trying to interfere with someone’s life. I wouldn’t know what to say or do if I was concerned about a farming mate who seemed down.*” (1FM03).

“*….I would not go to someone or an organisation who I haven’t had contact with before or who I have never met.*” (1FM03).

There was a broad agreement that people in general find the conversation on MH difficult:

“*Well I don’t think that the vast majority of people are comfortable broaching the subject or bringing it up because they don’t know what to do and they’ve not had any training in how to handle it.*” (1FM05).

A number of opinions were identified, relating to the type of person offering help and support:

*“Not really but I think if you’ve got some kind of knowledge, you don’t need to be from a background, it might help in some cases but no, you just need to be somebody that’s approachable.” (1FM09)*.

*“And, you know, the sort of hippy social worker type coming around is not going to find it easy, I’m being stereotypical here but they’re not going to find it easy to get that respectful relationship going, especially if farmers are bogged down in their own little kingdom and they’re not getting out much.” (1FM15)*.

The interviewees expressed that it was important to them, for people attempting to approach and engage IRFC to be relatable and approachable. It was also important to interviewees for the person to know about farming, however, the most desirable feature appeared to be approachability.

##### *Theme 4.3: religion*

Many farmers are members of local church communities and it was suggested that the church could play a role in supporting the wellbeing of farmers.

*“Another thing I’d say is that in England, a lot of support is delivered through the church or other organisations affiliated with the church.”* (1FM10).

*“I’ve suggested it to our church group that we have a dedicated person within our presbytery just calling on farmers day in/day out. It doesn’t have to be much, you could do about 20 calls a day quite easily ‘hello, how you doing, I’m so and so, we’re not going to shove anything up your nose or down your throat or anything like that, but is there anything we can do?’ and that’s what’s needed, it’s somebody calling.”* (1FM06).

##### *Theme 4.4: normalising mental health issues*

Participants shared the need for people to be able to speak more openly about struggles with mental health. It was felt that having high-profile people speaking out about their experiences would also help a culture shift, including case studies of people and farms and their stories. Farming organisations such as Young Farmers and RSABI were seen to be vital in promoting discussions about mental health.

*“Having people to share their personal stories and experiences and how they sought help, what personal barriers they had to overcome to get the point where they could ask for help and how getting the help impacted and supported them.*” (1FM10).

Participants did strongly feel that encouraging discussions about mental health should not happen unless the support was in place to help farmers who had made the big step to come forward.

“*Probably just that, just make it so routine that it’s not a stigma to talk about it. But I would say just more sort of coverage or presence of the fact that, yeah, mental health issues are there in the agricultural sector and there’s also a support network available.”* (1FM14).

They did not see this support as necessarily coming from health professionals, but also from bank managers, vets, accountants etc., who may see farmers more regularly.

*“Most farmers have a review with the bank manager, most farmers meet their vets several times, most farmers meet their accountant once or twice a year, you know, you can go 20 years without seeing your GP but you’ll see your accountant a couple of times a year. So maybe we’re looking at it the wrong way round [laugh], maybe it’s other professionals, not necessarily health professionals who should be helping?”* (1FM20).

One participant did highlight the problem that removing stigma around mental health issues can go too far in the opposite direction and lead people to think any down day was a symptom of a wider mental wellbeing issue.

*“I think that’s it, and I know the stigma’s better and I think on a sort of personal level I do worry that we often think of a down day as something to do with your mental wellbeing, but you’re actually meant to, you know, you do have days where you’re a bit low, it doesn’t mean you’ve got a mental health, it’s whether you’re low or, and stress is healthy as well, well not stress but pressure’s a bit healthy but if it goes on for too long it becomes stress; so it’s not all so… you know, stripping away the fact that it’s okay to feel different every day, but it’s just when you’re down or low for a long time that’s when it is, and I worry that there’s almost like an impact on you, not that you should be having mental wellbeing but almost like ‘ocht I can’t be bothered today’, that’s not a mental wellbeing issue, but if you feel like that for the next five days maybe you need to be thinking about it, but it’s okay for a day.”* (1FM19).

##### *Theme 4.5: approaching the conversation*

Participants reported that for farmers it is often normal to work long hours, often alone, so it can be difficult to recognise when low mood might be slipping into something more troublesome. The most valuable tool was friends, family, and other professional contacts keeping an eye out for mood changes and other symptoms.

*“Well it takes an awareness of others to recognise the changes in them and that requires the people around them to be clued up on it as well and be able to know how to react and how to signpost and that goes down to, you know, your vets visiting, your surveyors visiting and maybe them being trained to be able to or mental health first aid trained to be able to recognise the signs and be able to support, because you’re right, if farmers are working 24 hours for seven days a week they won’t see the small changes until suddenly they’re big changes.”* (1FM18).

Farmers also have to keep on working even when they are feeling down, so this may mask some of the symptoms of mental health problems until people are struggling much more.

*“So they’re still having to get on with the work whilst they’re either grieving or going through these issues, you know, if they’ve got mental health issues the cows have still got to be milked.”* (1FM05).

Some useful initiatives were highlighted, such as meeting farmers at marts as part of general health checks.

*“An organisation called ‘Fieldnurse’ has drop in clinics at some of the auction marts in Lancs where you turn up and there’s a qualified nurse there who will do your basic health check – cholesterol and all the other measurements that you do on a basic health check but they’ll also have a chat and they can understand – they’re either drawn from or can understand the farming community quite well so they’re the kind of people who are happy to have a chat and they can also make a referral – so people don’t have to make an appointment and go in to town or to some doctors, they can go and see them when they’re at the auction mart in an environment that feels familiar– they don’t even have to take their wellies or overalls off.”* (1FM10).

#### Theme 5: training

##### *Theme 5.1: mental health training for supporters of the farming community*

Those who regularly visit farmers such as vets, sales people, HSE inspectors etc. were seen as well placed to be aware of symptoms of mental health and how to approach farmers. Interviewees felt that this was currently on a fairly informal basis, although some mentioned the growing appetite for more formal mental health first aid training.

*“Yeah. I think we are placed well to raise it with farmers because we are speaking to them on a regular basis but we are probably not as it stands at the moment the best people to be doing that because that’s not where our strengths as a profession lie, we’re more practical than that and that’s where the training would fit in, so rather than find somebody who is well trained to deal with the situation, try and find a way for them to meet more farmers, it maybe makes more of a sense that people who are seeing farmers are receiving a little bit of training to spot warning signs and to raise the subject with farmers.”* (1FM04).

##### *Theme 5.2: health & safety, and mental health training*

Respondents were aware of the health and safety dangers on farms, especially for farmers who are working alone or are very isolated. The mandatory health and safety training, which is already part of many farming skills was seen as an ideal time to bring in aspects of mental health as well. In this way people would have had time to learn about symptoms and discuss the implications of mental health issues. The decline of HSE Health and Safety Awareness days was seen as a shame, as this was often a good time for farmers to discuss different farm-based scenarios and could have been a useful setting for mental health awareness-raising.

*“I think one way that you can get to these people and also to the farmers is health and safety. I think you want to make a course mandatory that they’ve got to go on and perhaps get a certificate to say that I’ve been on the course to understand or recognise symptoms of mental health problems; I really do think that’s about one of the only ways you’re going to get through, and make it part of the same thing as the chain saw people, you know, you have to do a refresher every three years, it only needs to be half a day but that is where you’ll meet up the tractor drivers or the livestock people will meet up and they will recognise because they’re talking about tractors or they’re talking about livestock, they will recognise within that group and they’ll start talking then.”* (1FM16).

Respondents felt that dedicated mental health training would be unlikely to be well attended, and that the best idea would be to build bits of mental health awareness into health and safety or crop/animal rearing training. There was also an understanding that farmers needed to be given training in a way that was useful for them, for example recognising additional learning needs.

*RES: That’s it, that’s exactly it and if it works I’ll be happy to look at that as ‘I know you maybe don’t want counselling but can I send you to this Live Life to the Full and maybe try some of that to see how you get on with that?’ and the thing is to remember, and I don’t know if that’ll matter, but dyslexia is quite high in farming.*

*INT: Oh good point.*

*RES: It’s about 10% of the population and 25% of farming.* (1FM19)

#### Theme 6: personal stories and experience

##### *Theme 6.1: who: individuals, organisations, & companies*

Interviewees talked about individuals and organisations who they had seen sharing information about mental health in farming or who could offer support for farmers. Others praised companies who had started to include mental health awareness in their employee training.

##### *Theme 6.2: what: examples*

Various examples of ways to help mental health were given by respondents, including things like areas setting up food banks which also helped to tackle rural wellbeing. Some also talked about training or mental health interventions they had previously received and which they found to be useful.

*“I don’t know if you’re aware, I won’t mention names but there is an almost retired farmer in this area who has an email group and he invites people round to his place once every so often for a coffee and a blether which I think is fantastic, but it’s mostly retired farmers, but if you’re working you’re not so likely to make the time to go. But you know, something like that when you are meeting with people that you’re already comfortable with is probably good, but there’s fewer opportunities for that.”* (1FM15).

##### *Theme 6.3: how: case studies*

Using case studies of other farmers was seen as a positive way of reaching farmers who often like to receive help and support from others in their own community. This was also seen as a way of normalising mental health issues and that others in the same situation were able to speak out about their mental health issues.

*“One of the things I have found – anonymous case studies work very well. If you can get those into the farming press, or into some sort of media they may be reading at home so if they have switched off from going to farmers’ meetings or attending community events, they may still be reading local newspapers or the farming press so if you can get case studies from farmers who are willing to talk about their own mental health issues and get that out in the public domain, I find that’s a useful way of trying to reach those folk.”* (1FM11).

##### *Theme 6.4: anecdotes*

Personal stories both highlighted the difficulties that farmers face, and gave suggestions for how best to contact farmers for a mental health intervention. Many of the interviewees had examples of famers who had died by suicide, often without those around them being aware of any previous mental health problems. Respondents felt that using case studies of personal stories could be beneficial to other famers in knowing they were not alone in some of the problems that they face.

#### Impact on plans for future pilot RCT

As a result of the findings of this study, the recruitment plan and proposed intervention for the pilot RCT has been adapted as shown in Table 3.

**Table 3:** Changes proposed to recruitment and intervention for a future pilot RCT

Change	Reason
- LLTTF made significant changes to the content of the farming specific modules.-	- To tailor it towards the farming community in response findings of the qualitative research findings.
- Paper copies of the LLTTF intervention made available.	- To allow an alternative format for those who experience poor internet connectivity or problems with technology.
- Standardised questionnaires used at baseline and at several subsequent points during the period of follow-up.	- To help farmers reflect on their own mental health, as some report being oblivious to their declining wellbeing over time.
- Support offered alongside LLTTF.	- To address the problem of loneliness and isolation, and to provide support with technology and accessing the online modules.
- Practical support for farmers (such as help with completing forms and interpreting an information leaflet), is provided in our study by RSABI.	- For those who are struggling with paperwork or feeling trapped within farming life.
- **Images, leaflets etc.**	
- Same images used in leaflets and online promotion.	- To promote recognition and reinforcement which might cause people to become more familiar with the idea of the study.
- Leaflets and social media referencing ‘crofting’.	- To capture the demographic of crofters who may not identify with the label of ‘farmer’.
- The intervention is made available in both written and video format online.	- Modules in video format help to provide additional options for those who struggle with reading.
- Participant information sheet and consent forms are re-written to be understandable to a wide range of reading ages.	- To consider the high proportion of farmers who may have difficulty with reading. There was a struggle to balance the mandatory university requirements for informed consent with a format that was suitable for this audience.
- Videos of other farmers talking about mental health and wellbeing, to supplement written materials.	- To respond to suggestions of using case studies of others in the farming community and normalising mental health issues.
- Images of farmers, rather than of livestock.	- To reinforce the concept of farmers looking after themselves, not just their animals.
**Meeting farmers where they are**	
- Using social media for recruitment.	- To reach farmers who may use social media and also to overcome some of the difficulties of recruiting during COVID lockdowns.
- Leaving leaflets at petrol stations, veterinary practices, auction marts, agricultural solicitors, machinery suppliers etc.	- To target recruitment at places where farmers are likely to visit.
- Using church ministers and church offices as gatekeepers for recruitment.	- Acknowledging the importance of religious organisations to many in the rural community and targeting recruitment through these.
- Recruiting through the Scottish Women’s Institutes and sports clubs, e.g. curling clubs.	- Targeting specific sports and local community groups that are known to be popular with people in rural areas. Also to specifically target women.
- Recruiting through the Young Farmers Associations.	- To specifically target younger farmers or those who may recognise mental health issues amongst older friends and family members.
- In person recruitment at Livestock Auction Marts by members of the research team.	- Face-to-face contact is seen to be one of the most important aspects of recruitment in the farming community.

## Discussion

From interviews carried out with those in the farming community we identified six themes on understanding the impact of farming life on mental wellbeing; how best to approach and engage farmers for a mental health intervention; and personal stories and anecdotes.

Respondents discussed a number of issues unique to the farming population in causing mental health issues, including struggles to maintain a work/life balance, isolation and loneliness, financial aspects, and the unpredictable nature of working with crops and livestock. Interviewees also talked about the difficulties of technology and paperwork, a lack of training in people management, and that older male farmers are less likely to seek help for mental health problems.

Supporting farmers with mental health issues will require better engagement, including using appropriate wording, and farmers tend to turn to those in their own community for help. More widely there was a call for increased training to recognise and support mental health issues, for example alongside health and safety training.

### Strengths and limitations

The strength of this study was the in-depth qualitative findings about mental health in the farming community and how best to approach farmers regarding the topic of mental health. A limitation is the low number of interviews with actual farmers, rather than those who work with farmers, however this was an unfortunate consequence of the change in methods required by the COVID-19 pandemic. For example, whilst those working with the farming community identified the difficulty in approaching farmers due to the potential for lost business we do not know if farmers themselves feel this way. Interviews with those who worked with farmers do have an advantage in discussing methods of approach already tried and tested by other organisations with a wealth of experience engaging with those in the farming community. Consideration could also be given of existing public health measures aimed to reduce the stigma associated with mental illness, potentially adapted to farming context. Although this study has focused on individual level factors, it is important to note the potential impact of wider societal factors such as policy gaps, under-investment in services, and the wider support infrastructure available in rural areas.

### Comparison with existing literature

The findings from our interviews with those in the farming community in Scotland show clear confirmation with other studies both in the UK and worldwide. The difficulties and unpredictability of farming life, together with feeling trapped within farming due to housing/family ties was key in both the literature and from our respondents [3].

Other studies have also found farmers are more likely to turn to their own communities for support than to health or social work authorities, with many preferring to engage with advice from respected members within their communities (such as vets) [18]. Whilst our respondents agreed with supporting farmers, those in wider farming businesses [18]. Whilst our respondents agreed with supporting farmers, those in business also found a barrier to discussing mental health was the risk of offending the farmer and therefore losing a business client. This was also found by JM Rudolphi and KL Barnes [10] who looked at using agribusiness as a way of supporting farmers’ wellbeing and found a reluctance to risk the business/client relationship.

There was agreement that ‘mental health’ was not the best terminology to use when engaging farmers, and that words such as ‘wellbeing’ were likely to be better received. This was also found by Rudolphi et al. (2019) in their study at an agricultural show in the USA, where agribusinesses were more comfortable using words that described symptoms, such as ‘depression’ or ‘anxiety’, rather than ‘mental health’. Another approach is to use more colloquial words or farm-related metaphors, as found by Davies et al. [11] and also reported by our respondents.

The use of anonymous supports such as the internet or self-help booklets was not raised as much in our findings as in other studies [18]. Our respondents did, however, discuss in some detail the struggle that many farmers face with technology, paperwork, and reading abilities, including the problem of dyslexia. Whilst anecdotally there appears to be a high prevalence of dyslexia within the farming community we couldn’t find any supporting literature on the prevalence rates, although one report indicated that the rates are higher than in the non-farming community [26].

MC Bondy and DC Cole [14] and DC Cole and MC Bondy [5] refer to ‘meeting farmers where they are’ in an effort to bring public health to the farming community. Our respondents also highlighted various methods for recruitment found in other studies such as leafleting places were farmer are likely to be, including petrol stations and targeting sports clubs and religious organisations [3, 13]. Having a famous person in the community, or equally a local person known to others, speaking about their mental health was reported to increase normalisation of mental health issues and give farmers somebody to identify with [16].

Respondents recognised that mental health in the farming community was an important issue to tackle, but also that the farming community could be a difficult group to reach and engage. This study has highlighted the importance of farmers seeking help within the farming community and of ‘meeting farmers where they are’ in approaching farmers for recruitment.

## Conclusions

Our research has highlighted a number of important issues in the recruitment of farmers for a mental health intervention, including the importance of wording and accessibility to those with poor internet or difficulty with reading. Meeting farmers where they are was found to be a key message for recruitment, as well as wider public health delivery to those in the farming community.

Our research has also led to adaptations of a potential on-line intervention so that it includes more tailoring of content, more options for accessibility and options for personal/guided support.

Our findings have important practical implications for others looking to recruit farmers to intervention studies. Further research is needed on the acceptability and feasibility of intervention(s) and whether the suggested methods for recruiting farmers are acceptable in practice, alongside other information on intervention study retention/attrition and outcome completion rates.

## Electronic supplementary material

Below is the link to the electronic supplementary material.

Supplementary Material 1 Topic Guide

## Data Availability

The datasets generated and/or analysed during the current study are not publicly available due the sensitive nature of the topic (suicide prevention in small communities where participants might be identified) but are available from the corresponding author on reasonable request.

## List of abbreviations

RCT: Randomised Controlled Trial
MH: Mental Health
