# Supplementary material for: Engaging the agricultural community in the development of mental health interventions: a qualitative research study
Source: BMC Psychiatry. 2023 Jun 5;23:399. doi: 10.1186/s12888-023-04806-9 (PMC10240118; doi:10.1186/s12888-023-04806-9)
Supplement: Supplementary file 1 — Supplementary Material 1 Topic Guide [file 12888_2023_4806_MOESM1_ESM.docx]

**Supplementary Information**

Topic Guide

1. What are the best ways of reaching people in the farming community with support options for their mental health?
2. What would help farmers to recognise when they might benefit from help?
3. Can you suggest wording that individuals might find acceptable / easy to use to talk about
   1. Poor mental health,
   2. Support for mental health?
4. What could be done to encourage individuals to seek help?
5. What might discourage or prevent individuals from seeking help?

What might make individuals stop receiving the support they need?
